# Complete Genome Sequence of *Escherichia* Phage ADB-2 Isolated from a Fecal Sample of Poultry

**DOI:** 10.1128/genomeA.00043-13

**Published:** 2013-03-14

**Authors:** D. V. Bhensdadia, H. D. Bhimani, C. M. Rawal, V. V. Kothari, V. H. Raval, C. R. Kothari, A. B. Patel, V. D. Bhatt, N. R. Parmar, M. R. Sajnani, P. G. Koringa, C. G. Joshi, S. P. Singh, R. K. Kothari

**Affiliations:** a Department of Microbiology, Christ College, Rajkot Gujarat, India; c Department of Biotechnology, Christ College, Rajkot Gujarat, India; b Department of Biosciences, Saurashtra University, Rajkot Gujarat, India; d Department of Animal Biotechnology, College of Veterinary Science & Animal Husbandry, Anand Agricultural University, Anand, India

## Abstract

*Escherichia* phage ADB-2 was isolated from a chicken fecal sample. It is a virulent phage and shows effective inhibition of *Escherichia coli* strains. Here we announce the completely sequenced genome of *Escherichia* phage ADB-2, and major findings from its annotation are described.

## GENOME ANNOUNCEMENT

*Escherichia coli* is an opportunistic pathogen in poultry. With the rapid emergence of antibiotic-resistant bacteria, the use of bacteriophages has regained attention as an efficient alternative method for their control (1, 2). Virulent phages cause bacterial host cell lysis and not only function to control bacterial populations but also can be used as indicators of bacterial (fecal) contamination (3, 4) and as tools for identifying (typing) specific bacterial strains (5, 6). Poultry meat is one of the most popular foods. Poultry and poultry meat are often found to be contaminated with potentially pathogenic microorganisms. Improvements in biosecurity on poultry farms are likely to be very expensive and difficult to maintain (7), so there is a need to find an acceptable, cost-effective way of preventing infection of poultry with coliform bacteria (8).

*Escherichia* phage ADB-2 was isolated from a fecal sample of poultry by the double-layer agar plaque method. The Spot test (9) and DAL method were used to determine the host range of the *Escherichia* phage ADB-2. An antibiogram of the natural host of *Escherichia* phage ADB-2 showed that the host was sensitive against norfloxacin and gentamicin and that it demonstrated higher resistance against cotrimoxazole and oxytetracycline (data not shown). *Escherichia* phage ADB-2 was purified by ultracentrifugation and by the CsCl_2_ density gradient purification method. Genomic DNA was extracted from the stock by the alkaline lysis method. The whole-genome sequencing of *Escherichia* phage ADB-2 was performed using Ion Torrent PGM (Ion 200-bp sequencing kit) (Life Technologies).

The data generated from the genomic library contained 229,781 reads and 45,496,800 nucleotide bases with average read length of 198 bases. The assembly using Newbler version 2.6 generated a 50,552-bp-long single chromosome. The genome annotation and comparative analysis of the genome were done using Rapid Annotation using Subsystem Technology (RAST) (10). The phage has 46% GC with 76 predicted coding regions and 2 RNA genes.

This genome contains functional genes related to phage structure and packaging machinery (major capsid protein, unknown phage structure proteins, and terminase), phage neck protein, tail structure for host interaction (tail fiber protein, tail sheath protein, and tail-associated protein), phage DNA synthesis (helicase, DNA-directed RNA polymerases, endoDNase, and transcription regulator) and host lysis (endolysin without holin). These functional genes are scattered over the genome. The complete genome analysis of this phage provides new insight into its characteristics and interactions with *Escherichia coli*.

### Nucleotide sequence accession number.

The complete sequence of the *Escherichia* phage ADB-2 genome can be accessed under the GenBank accession number JX912252.1.
